# Redefining the Wash Resistance Index to predict insecticidal durability of mosquito nets

**DOI:** 10.5281/zenodo.18461060

**Published:** 2026-02-02

**Authors:** Ole Skovmand, Duoc M Dang, Trung Q Tran, Rune Bosselmann, Sarah J Moore

**Affiliations:** 1 VectorisIQ SARL, Castelnau le Lez, France; 2 Biolytrics Laboratories, Hanoi, Vietnam; 3 Faculty of Pharmacy, East Asia University of Technology, Hanoi, Vietnam; 4 Vegro Aps, Copenhagen, Denmark; 5 Vector Control Product Testing Unit, Environmental Health and Ecological Sciences, Ifakara Health Institute, P.O. Box 74, Bagamoyo, Tanzania; 6 New Vector Control Interventions Group, Swiss Tropical & Public Health Institute, Kreuzstrasse 2, 4123 Allschwil, Basel, Switzerland; 7 Faculty of Science, University of Basel, Petersplatz 1, 4001, Basel, Switzerland

## Abstract

**Background:**

Insecticide-treated nets (ITNs) are a core malaria vector control intervention recommended by the World Health Organization (WHO). To obtain WHO endorsement, ITNs must demonstrate sustained bio-efficacy after repeated washing through standardised laboratory and semi-field evaluations. Wash resistance (WR) is typically evaluated over 20 washes (WR20) to approximate three years of use. Routine WR20 testing is impractical for quality control. To address this, the Collaborative International Pesticides Analytical Council (CIPAC) introduced the Wash Retention Index (WRI), based on four consecutive daily washes. The WRI was defined as the fourth root of the ratio of insecticide content after four washes to the initial content and adopted as a quality-control specification. This article examines the WRI and the prediction of WR20 when including or excluding the first wash with the aim to define a WRI method that more accurately reflects WR20, while remaining practical. Because surface insecticide may increase during storage due to diffusion from the polymer, the effect of excluding the first wash from index calculations to reduce storage-related variability was assessed.

**Materials and Methods:**

WR data were extracted from WHO Pesticide Evaluation Scheme (WHOPES) reports, and WRI values calculated assuming 1-day washing intervals. Additional data were generated from polyester and polyethylene nets with insecticide content quantified.

**Results:**

When WR20 was regressed on WRI (including the first wash), the model fit was weaker and significant effects of insecticide chemistry and polymer were retained, indicating that WRI does not capture intrinsic wash-off kinetics. In contrast, new WR (excluding the first wash) explained more variance in WR20 (adjusted R^2^= 0.76 vs 0.71), with a slope approaching proportionality (0.66 vs 0.31) and with no residual effects of chemistry or polymer.

**Conclusions:**

Excluding the first wash from WRI improves prediction of WR20. First-wash loss reflects production and storage effects rather than intrinsic wash resistance. Wash resistance can be more intuitively expressed as the Wash-Off Rate Index (WORI), representing average wash-off between washes 1-5.

## Introduction

Malaria remains a major global health challenge and is transmitted by mosquitoes of the *Anopheles* genus. The majority of malaria cases occur in Africa, where the dominant *Anopheles* species bite humans primarily indoors at night [1]. As a result, sleeping under insecticide-treated nets (ITNs) is an effective preventive measure. To date, more than 3 billion ITNs have been distributed globally with around half of those at risk of malaria in sub-Saharan Africa sleeping under an ITN [2].

Since around 2000, most ITNs have been manufactured as long-lasting insecticidal nets (LLINs), designed to retain efficacy after repeated washing. Two principal technologies are used: (i) insecticide applied as a surface coating to polyester (PET) nets using a foulard process, and (ii) insecticide incorporated into polyethylene (PE) prior to extrusion into yarns that are subsequently knitted into nets [3]. In both systems, the majority of insecticide is contained within the polymer matrix, with a smaller fraction present at the surface either immediately after manufacture or through subsequent diffusion [4].

Loss of surface insecticide through washing, evaporation, or abrasion is offset by diffusion from the polymer interior, a process termed regeneration. The regeneration time (RT) is defined as the period required for surface insecticide to return to equilibrium. Historically, RT has been estimated using mortality bioassays with pyrethroid susceptible mosquitoes [5,6]. However, bioassays may reach 100% mortality before physicochemical equilibrium is achieved [4], leading to systematic underestimation of true RT.

Consequently, most ITNs were historically assigned an RT of 1 day. This was supported by tests showing that nets washed 3 times within a single day still produced full mortality in fully susceptible mosquito strains the following day. Assuming a RT of 1 day, wash resistance (WR) was then modelled using total insecticide content measured at baseline and after 1, 2, 3, 5, 10, 15, and 20 daily washes (WR20), typically applying an exponential decay function based on the free migration model [7]. Wash resistance is the ability of an insecticide-treated net to retain sufficient biologically active insecticide following a specified number of standardised washes under controlled conditions to meet predefined efficacy criteria, as measured by mosquito mortality in standard bioassays (traditionally 80% mortality of pyrethroid susceptible mosquitoes within 24 hrs). This artificial aging method is used to simulate an ITN that has been used for three years based on field and laboratory data for pyrethroid ITNs [5,8,9]. Data were further explored and shown to broadly align with bioassay data [7].

Although WR20 measurement is a useful indicator of ITN chemical durability for pyrethroid ITNs, it is time-consuming and impractical for routine quality assurance. To address this limitation, the World Health Organization Pesticide Evaluation Scheme (WHOPES) and Collaborative International Pesticides Analytical Council (CIPAC) introduced the Wash Retention Index (WRI).

The WRI is a laboratory-based metric that quantifies the proportion of insecticide retained on or within an insecticide-treated net after a defined series of washes, expressed relative to the initial insecticide content and intended as a rapid surrogate measure of WR20. It is an accelerated method in which nets are washed daily for 4 days, with insecticide content measured at day 0 and day 4 [10,11]. Nets are stored at 40 °C between washes, and WRI is defined as the fourth root of the ratio of insecticide content at day 4 to that at day 0. This protocol differs from WR20 testing, where nets are stored at 30 °C and washed at intervals defined by the RT. Despite its intended purpose, the original WRI frequently failed to predict WR20 reliably. In some cases, manufacturers reported wide WRI ranges, corresponding to insecticide losses of up to 20% per wash, which are inconsistent with typical ITN performance.

Several WHOPES studies measured both insecticide content and bio-efficacy (cone bioassays) before washing and after 1, 3, 5, and subsequent washes up to 25 [5,11]. These datasets allow derivation and comparison of WR20 (20 washes), WRI (4 washes), initial surface concentration (0–1 wash), and wash-off rates (1–5 washes). The present study builds on these published data and supplements them with additional data from experimental nets to examine the effects of (i) washing method on PE and PET nets and (ii) denier of coated ITNs on WRI.

The objective of this study was to define a WRI method that more accurately reflects WR20, while remaining practical for routine ITN characterisation. Because surface insecticide may increase during storage due to diffusion from the polymer interior, we assessed the effect of excluding the first wash from index calculations to reduce storage-related variability.

## Materials and Methods

### Extraction of WHOPES Data

Data on the wash resistance of commercial ITNs were extracted from WHOPES reports published between 2003 and 2017 [12-17]. Nets were included only where both chemical analyses and bioassay results were available for the same product. From these datasets, wash resistance (WR), Wash Retention Index (WRI), wash-off rates, and estimates of surface concentration were derived. WHOPES reports typically provide total insecticide content after 3 and 5 washes. Insecticide content after 4 washes was therefore estimated by linear interpolation, calculated as the mean of the values at 3 and 5 washes [7].

### Experimental work with net materials

**Polyethylene (PE) nets**: Insecticide-incorporated nets were produced at the WHO-Prequalification (PQ)–audited Sunpack factory (Guangzhou, China). Yarns were extruded from blends of high-density (HDPE) and low-density polyethylene (LDPE). Yarns contained deltamethrin (DM) at 2.0 or 14 g/kg, with optional piperonyl butoxide (PBO) at 1.7 g/kg; a PBO-only yarn (16.3 g/kg) was also produced. Yarn diameters ranged from 0.13 to 0.14 mm.

The two yarn types were knitted together into fabric. To assess insecticide release without interaction effects, additional fabrics were produced using each yarn type alone. Yarn batches were colour-coded for identification. Each knitting machine produced five parallel 30-cm-wide fabric bands; each band was treated as an independent sample. Test pieces were cut across the full width of each band, with length determined by analytical requirements.

**Polyester (PET) nets**: Insecticide-coated nets were manufactured by knitting extruded yarns into fabric, followed by insecticide coating using a foulard process [3]. After coating, fabric was cut and sewn into finished nets. In PET nets, heterogeneity across the fabric width arises from pressure variation in foulard rollers, while heterogeneity along the length reflects application dynamics of the coating solution [3].

Experimental PET formulations containing 2g DM/kg net were produced at factory scale at Moon Netting (Lahore, Pakistan). Four nets with different yarn diameters but identical coating formulation and dosage (g/m^2^) were tested. Additional PET samples (30 × 30 cm) were prepared using a laboratory foulard (Ernst-Benz, Zurich, Switzerland) and dried in a laboratory oven (Mini Thermo, Roaches, USA).

### Sampling

Two sampling protocols were applied:

**WHOPES Sampling Protocol**: Following WHOPES guidance [6], five samples were taken from each of three nets: four diagonally across the side panels and one from the roof. This protocol was applied to the four PET nets with different yarn diameters. From each sample, a circular cut was made of 100 cm^2^, which was cut into small pieces, thoroughly mixed, and three subsamples were weighed. Each subsample was analysed in triplicate at Biolytrics laboratories, Hanoi.

**Figure 1:**
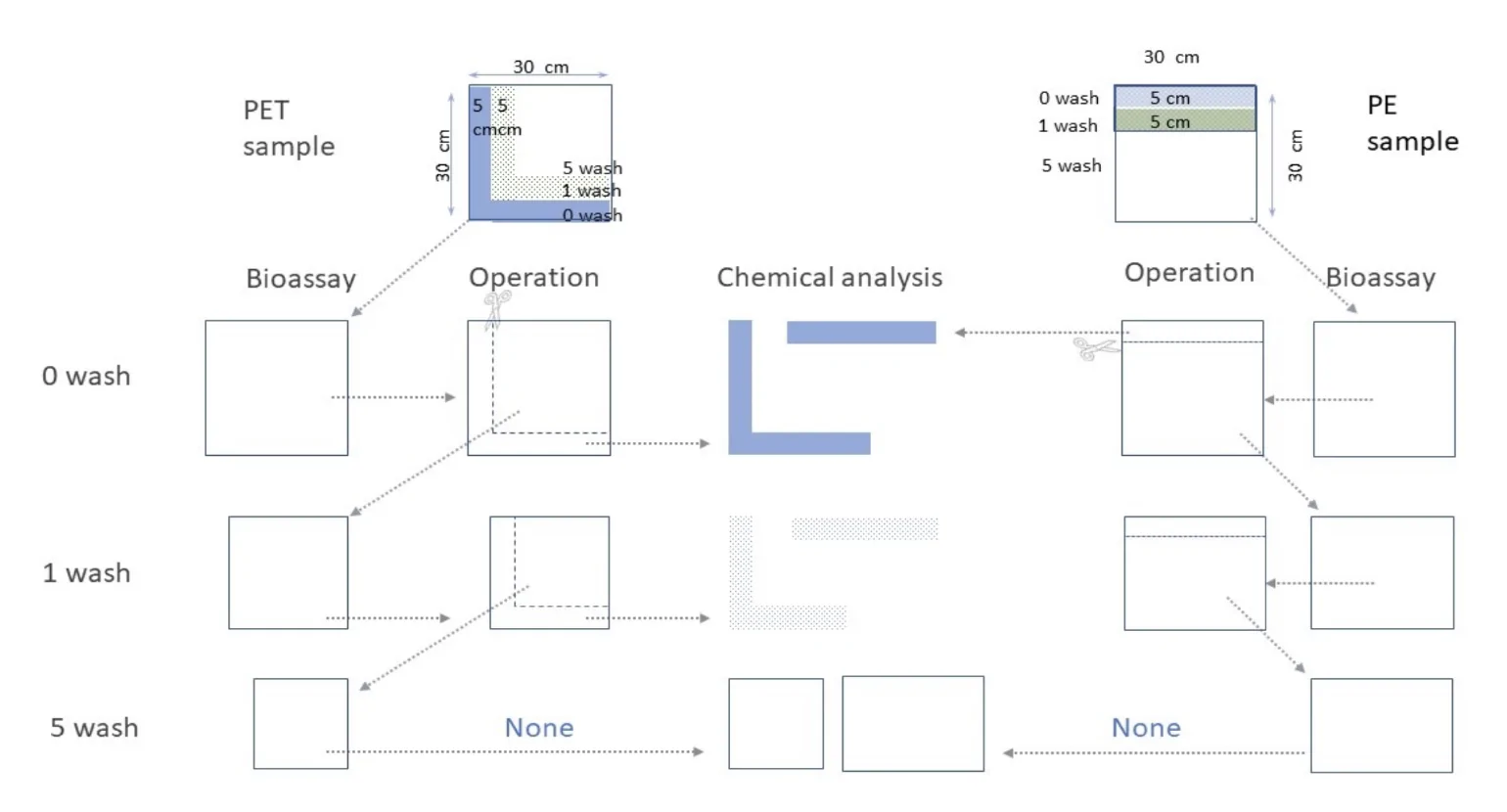
Subsampling method for minimising pre-wash variation. Subsamples were cut from 30 × 30 cm net sections in a way that minimised variation in pre-wash dosage between samples analysed after 0, 1, and 5 washes. For PET nets, L-shaped subsamples reduced heterogeneity, whereas for PE nets, top-edge sub-samples were most consistent.

**Sampling for Experimental Studies**: For studies assessing soap type, pre- and post-wash samples were cut from the same fabric piece to minimise heterogeneity (Figure 1).

For PET nets, samples were taken along the fabric length. Subsampling involved cutting an L-shaped section from one corner to capture both warp and weft yarns. The 5-cm L-section was used for chemical analysis, with the remaining fabric used for bioassay (Figure 1).

For PE nets, the same swatch was used for chemical analysis and bioassays: the upper 5 cm was removed for chemical analysis, and the remaining 25 × 30 cm was used for post-wash chemical analysis, bioassays, or both. The use of the upper 5cm was compared with the L-shaped sampling method used for PET in one experiment.

### Wash method

Most WHOPES datasets were generated using the pre-2013 CIPAC protocol, in which 25 × 25 cm swatches were washed in 1 L glass bottles containing 500 ml 5% unscented Marseille soap and agitated for 10 minutes in a shaking water bath at 30 °C [5].

In this study, 25 × 25 cm swatches were washed in 1 L glass bottles containing and agitated for 10 min in a shaking water bath at 30 °C. The bottle-shaker method was used with the CIPAC-defined soap [6,10] and the 5% unscented Marseille soap tested in parallel on PET and PE nets to assess the effect of soap type.

### Chemical analysis

For WR determination, nets were washed and analysed at 0, 1, 3, and 5 washes, and subsequently up to 20 or 25 washes using intervals defined by the WHO regeneration-time method [6]. Details of chemical analytical methods are detailed in the corresponding ITN product specifications. Samples from nets examined in this study were extracted with xylene under reflux, the extract filtered and corrected to volume and injected into Gas Chromatograph (GC-FID 7890 Agilent) using dipropyl phthalate as internal standard [4].

### Calculation of WRI

CIPAC WRI [6] was here calculated as a percentage using baseline insecticide content and the mean content after 3 and 5 washes (equation 1, where n=number of washes, t = total active ingredient (AI) in g/kg after 4 washes (t_4_) or unwashed (t_0_), allowing derivation from WHOPES datasets despite the absence of reported WRI values. As most nets were washed at 1-day intervals, these data align with the original CIPAC WRI design.

(1) 100  × t4/t04

The New WRI was calculated using insecticide content after 1 and 5 washes (equation 2), based on both WHOPES data and experimental datasets generated in this study.

(2) 100  × t5/t14

### Use of WOR and WORI

The wash-off rate (WOR) quantifies insecticide loss per wash from an ITN, calculated from changes in chemical content following defined washing intervals, and provides a direct measure of insecticide removal rather than retention. For clarity, hereafter retention-based metrics are expressed as wash-off metrics as this is more relevant when considering ITN chemical durability:

WOR (Wash-Off Rate) = 1 − WR

WORI (Wash-Off Rate Index) = 1 – WRI

This representation makes differences more intuitive. For example, WOR values of 1% and 4% clearly indicate a four-fold difference in wash-off, whereas WR values of 99% and 96% may appear similar. WHO has recognised that WOR correlates with surface insecticide concentration [18].

### Statistical analysis

Analysis was conducted in STATA 19 (Stata Corp, Texas). Statistical significance was defined at α = 0.05.

### Secondary data analysis

**Effect of including or excluding first wash**: Assuming a model of exponential insecticide loss [7], the wash retention index (WRI) was calculated by taking the “nth” root of post-washing insecticide content divided by the pre-washing content. Two indices were made the CIPAC WRI that is 0 to 5 washes (equation 1) and the new WRI which excludes the first wash (equation 2). Exponential curves were then fitted to all wash data up to 20 washes to estimate the retention index at each wash. Based on these data, the average retention index was calculated (EXP20). The average retention over 20 washes was regressed against either CIPAC WRI or New WRI with insecticide and polymer added as categorical variables.

**Effect of polymer and insecticide**: Using the exponentiated 20 wash off estimates for all ITNs, a gamma log-link generalised linear model was run with insecticide and polymer interacted as categorical variables to explore the effect of polymer. Separate models stratified by polymer were run to investigate the effect of insecticide because the design was an incomplete factorial (PE contained PBO and permethrin while PET contained chlorfenapyr), therefore polymer-stratified models were used to avoid extrapolating to non-existent insecticide–polymer combinations. Model assumptions (data structure and link) were evaluated through applying the link test to confirm adequate link function specification and inspection of residuals and fitted values.

### Analysis of experimental data

**Effect of Soap Formulation**: Wash retention indices (WRI) for DM and PBO were separately analysed using a linear mixed-effects model with wash protocol (Marseille vs CIPAC) specified as a fixed effect and individual nets included as a random intercept to account for repeated measures. Model fit for the linear mixed-effects analysis was checked using residual diagnostics (standardised residuals, residual–fitted value plots) and inspection of the random intercepts to verify that assumptions of normality and homoscedasticity were reasonable. Net 26 was removed as it was an outlier and its removal substantially improved model fit.

**Comparison of WRI by denier**: Wash resistance was quantified as the percentage of active ingredient (AI) remaining after CIPAC standard washing at 1, 5, and 20 washes. Wash Retention Indices (WRI) were calculated using both the CIPAC method (CIPAC WRI) and the newly proposed New WRI and WOR metrics. For each denier class (75D, 100D, 150D), mean AI loss and WRI values were computed and expressed with standard errors. Because WRI values were not normally distributed and group sizes were unbalanced, differences in WRI between denier classes were evaluated using a Kruskal–Wallis equality-of-populations rank test.

## Results

### Analysis of Historical WHOPES Data

**Effect of including or excluding first wash**: WHOPES reported insecticide-retention curves for many ITN candidates tested between 2007 and 2017 [12-17]. These datasets, typically presented as total insecticide content after defined numbers of washes, describe an exponential decline in insecticide content under repeated washing. From these data, we calculated 1) the CIPAC WRI (and corresponding WOR 0-4), 2) the amount of AI lost on the first wash (WOR 0-1), and 3) the New WRI (average wash-off between washes 1 and 5, (WOR 1-5)), and 4) wash resistance (WR 20), estimated as the exponent of the decay curve over 20 washes. For clarity, all indices were expressed as wash-off metrics (WOR) and summarised in Table 1.

**Table 1:** Wash-off rates (WOR) calculated from WHOPES data (2003–2017) and additional Tsara Soft tests (2019).

ITN Name	Year	Polymer	Wash interval (days)	Insecticide	Dosage g a.i./kg	WOR 0-4	WOR 0-1	WOR 1-5	WR 0-20
Yorkool	2007	PET	1	DM	1,82	0,764	-4,400	1,895	0,167
Netto	2007	PET	1	DM	2,10	8,290	16,670	4,103	4,161
Hike	2007	PES	1	DM	1,75	4,022	4,571	4,143	2,339
Permanet 3 roof	2009	PE	1(5)	DM	4,66	1,338	2,135	1,073	0,602
Permanet 3 roof	2009	PE	1(5)	PBO	26,80	7,820	12,295	6,350	3,067
Permanet 2	2003	PET	7	DM	1,19	0,745	4,316	3,240	3,886
Veeralin		PE	5	AM	7,38	0,512	1,220	0,069	0,230
Veeralin		PE	5	PBO	2,60	1,525	1,930	1,606	1,607
DawaPlus 2	2009	PET	1(7)	DM	2,06	2,371	0,000	2,000	1,477
DawaPlus 3	2017	PET	1	DM	2,40	2,600	-2,083	3,365	3,365
DawaPlus 4	2017	PE	1	DM	2,44	0,257	0,414	0,616	0,116
DawaPlus 4	2017	PE	1	PBO	10,20	1,248	7,843	-1,049	1,067
Olyset Plus		PE	2	PERM	19,17	5,911	5,791	4,751	1,919
Olyset Plus		PE	2	PBO	9,17	9,131	18,181	6,059	3,236
InterceptorLN	2015	PET	1	AM	4,40	6,519	4,772	6,737	7,364
InterceptorLN	2017	PET	1	AM	5,62	13,543	32,918	7,167	9,152
InterceptorG2	2015	PET	1	AM	2,47	5,800	12,551	2,775	3,364
InterceptorG2	2017	PET	1	CLF	5,23	10,075	16,443	7,422	8,233
Miranet LN	2015	PE	2	AM	5,10	0,074	-0,784	0,342	0,144
Pandanet 2	2015	PE	3	DM	1,83	0,274	-0,546	0,414	0,413
Safenet	2015	PET	1	AM	7,50	6,836	15,733	4,668	3,047
Tsara Soft 75 D	2019	PET	1	DM	2,72	10,302	27,219	3,635	3,341
Tsara SoftlOOD	2019	PET	1	DM	1,87	9,518	22,617	3,639	3,547
TsaraSoftlOODF	2019	PET	1	DM	1,94	9,800	21,199	3,623	4,505
Tsara Soft 150D	2019	PET	1	DM	1,86	7,985	16,433	4,134	3,262

Data extracted from WHOPES reports for polyester (PET) and polyethylene (PE) nets containing deltamethrin (DM), alphacypermethrin (AM), permethrin (PERM), chlorfenapyr (CLF), and/or piperonyl butoxide (PBO). Wash intervals follow WHOPES evaluations; alternative intervals are shown in parentheses. WOR indices express percentage insecticide loss per wash relative to initial content. WR20 exponent of the decay curve over 20 washes representing average percentage insecticide loss per wash relative to initial content over 20 washes.

Across ITNs, WOR over 20 washes ranged from 0.1% to 9.2%, representing a 92-fold difference in intrinsic wash-off performance. First-wash loss (WOR ₀–₁) was consistently much higher than losses during subsequent washes, indicating that the first wash is disproportionately influential and does not reflect intrinsic wash resistance.

**Effect of polymer and insecticide**: When EXP20 was regressed on CIPAC WR (including the first wash), the model fit was weaker and significant effects of insecticide chemistry and polymer were retained (Table 2), indicating that CIPAC WR does not capture intrinsic wash-off kinetics. In contrast, New WR (excluding the first wash) explained more variance in EXP20 (adjusted R^2^= 0.76 vs 0.71), with a slope approaching proportionality (0.66 vs 0.31) and with no residual effects of chemistry or polymer. This suggests that first-wash loss reflects manufacturing/stability phenomena, whereas subsequent wash-off reflects true wash resistance relevant to long-term performance.

**Table 2:** Effect of polymer and insecticide on wash off indexes.

Model	Adjusted R2	WOR coefficient	t-stat	p-value	PET coef.	p-value	Deltamethrin coef.	P
WOR15	0.7550	0.662	4.32	<0.0001	+1.33	0.131	-1.05	0.114
WOR04	0.7137	0.312	3.66	0.002	+1.80	0.050	-1.34	0.060
WOR01	0.6539	0.096	2.82	0.011	+2.52	0.009	-1.48	0.057

Variance analysis of the WHOPES data in Table 1 and indexes for wash off show the significance of the impact of polymer and deltamethrin insecticide on the indexes. Deltamethrin is shown because it has the largest sample size PE n=3 and PET n=10. Coefficient PET is relative to PE and deltamethrin relative to alphacypermethrin.

**Effect of polymer and insecticide**: A gamma log-link GLM of the exponentiated 20x wash off (Table 3) demonstrated significantly higher wash off of insecticide from PET (coefficient 3.42, z= 8.17 p<0.001) than PE nets. When considered by polymer, PBO and permethrin showed >10-fold higher loss per wash than alphacypermethrin and 5-fold higher wash off than deltamethrin from PE nets. In PET nets chlorfenapyr wash off was higher but not significantly so, while deltamethrin wash off was lower. Polymer effects were large, with PET exhibiting substantially greater loss than PE. Together, results show that wash-off is both insecticide-specific and polymer-dependent. An additional regression of incorporated PE nets showed higher loss of permethrin and PBO than alphacypermethrin or deltamethrin and no lower loss of deltamethrin from coated PET nets (Table 3).

**Table 3:** Loss of different active ingredients from PE and PET nets using the WOR15 model.

	Polyethylene (PET) n=15			Polyester (PE) n=10		
Insecticide	coefficient	z-stat	p-value	coefficient	z-stat	p-value
Alphacypermethrin	1			1		
Deltamethrin	0.52	-2.36	0.018	2.01	1.47	0.141
Permethrin				10.26	3.64	<0.001
PBO				12.00	5.50	<0.001
Chlorfenapyr	1.44	0.70	0.485			

### Experimental validation using newly produced nets

**Effect of Soap Formulation**: Twenty industrial-scale PE nets were washed using bottle shakers with either unscented Marseille soap or the CIPAC-defined soap. From each net, three 30 × 30 cm samples were taken across the fabric width. L-shaped subsamples were removed for pre-wash chemical analysis; remaining fabric was washed four times at one-day intervals (Figure 1). CIPAC WRI values were calculated and averaged by formulation (Table 4).

**Table 4:** Effect of soap type on CIPAC WRI (%) for polyethylene (PE) nets. Polyethylene (PE) contained incorporated deltamethrin (DM) or piperonyl butoxide (PBO). Values are mean ± standard error (SE).

Net Polymer	N	Marseille soap	CIPAC soap	Coefficient	p-value
PE (DM)	10	100.04 ± 0.91	99.46 ± 2.50	0.580	0.501
PE (PBO)	10	95.48 ± 1.47	96.10 ± 1.75	-0.466	0.107

For PE nets, the choice of washing detergent did not affect insecticide retention. After 20 washes, deltamethrin retention was 100.04 ± 0.91% with Marseille soap and 99.5 ± 2.50% with CIPAC soap (p = 0.50). PBO retention was similarly comparable between detergents (95.5 ± 1.47% vs 96.1 ± 1.75%; p = 0.11). The data from this experiment indicate that Marseille and CIPAC soaps produced equivalent wash outcomes for PE nets within normal experimental variability. However, CIPAC soap was used for further experiments.

**Comparison of WRI by denier**: Twelve PET nets were made with yarns of each of 3 deniers (75, 100 and 150 denier (D)) and thus different diameters. Wash retention did not differ significantly by denier (Table 5). After 20 CIPAC washes, 75D nets lost 27.2% of deltamethrin, 100D nets lost 21.9%, and 150D nets lost 16.4% relative to baseline. CIPAC WRI (0.94–0.96) and New WRI (0.96 across all deniers) were consistent and showed close agreement. Most wash-off occurred between wash 0 and wash 1 (WOR 0–1: 16.4–27.2%), whereas subsequent losses to wash 20 were smaller (WOR 0–20: 3.2–4.0%). The New WORI that excludes the first wash off closely matched WOR, which reflects the average wash resistance over 20 washes. Although the pattern suggested lower cumulative loss in higher denier nets, differences were not statistically significant (Kruskal–Wallis χ^2^(2)=3.27, p=0.195).

**Table 5:** Effect of yarn denier on first-wash and 20-wash loss in PET nets.

Net Type	Start Dosage (g/kg)	After 1 Wash (g/kg)	After 5 washes (g/kg)	After 20 Washes (g/kg)	CIPAC WRI	New WRI	WOR 0-1 (%)	CIPAC WORI (%)	New WOR (%)	WOR 0-20 (%)
75 D	2.700 (±0.039)	1.976	1.704	1.161	0.936 (±0.026)	0.964	27.2	6.4	3.6	3.3
100 D	1.938 (±0.149)	1.487 (±0.021)	1.283 (±0.018)	0.689 (±0.019)	0.937 (±0.019)	0.964	21.9	6.3	3.6	4.0
150 D	1.839 (±0.045)	1.551	1.310	0.810	0.956 (±0.024)	0.959	16.4	4.4	4.1	3.2

Factory-coated PET nets with differing denier were washed 1 and 20 times and chemical content was measured. Wash off rate WOR–1 is the wash of rate over the first wash; WOR–20 is the average wash off rate over the 20 washes calculated from the percentage loss relative to start dosage. Values are mean ± standard error (SE).

**Figure 2:**
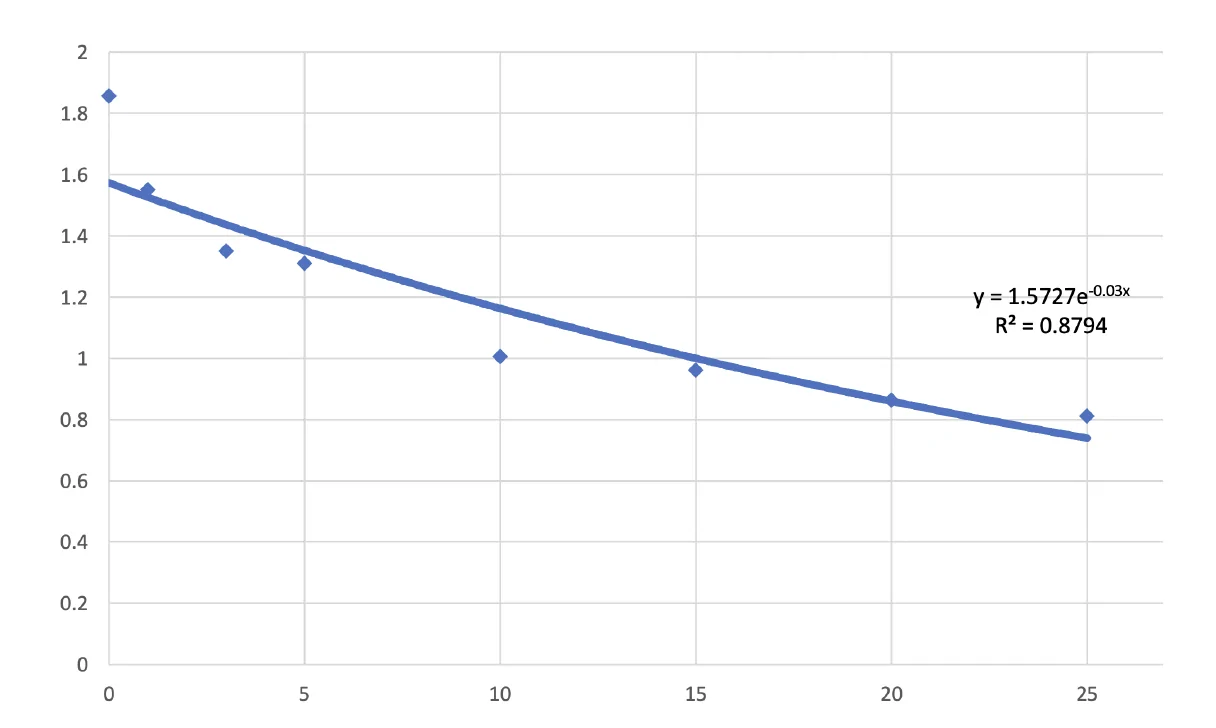
Exponential decay of deltamethrin in a PET (75 denier) net. A 75D PET net was washed according to WHO procedures, and total deltamethrin content was quantified after 0, 1, 3, 5, 10, 15, 20, and 25 washes. The initial (0-wash) dosage lies well above the fitted exponential regression line, indicating an unusually high first-wash loss.

From the secondary data analysis, coefficients of variation for New WORI and WOR were similar and substantially lower than those for WOR₀–₁ and the CIPAC WORI, indicating improved precision. Taken together with the data from experimental nets, these findings demonstrate that New WORI provides a more accurate and statistically efficient estimate of wash resistance, requiring fewer samples to detect differences. As illustrated for the PET 75D net (Figure 2), insecticide content declined exponentially with wash number (r^2^= 0.88), but the baseline (0-wash) value lay well above the fitted regression line. Similar patterns were observed across multiple WHOPES datasets.

## Discussion

The Wash Retention Index (WRI) was introduced by WHO in 2013 based on the CIPAC standardised washing protocol MT195 [10]. Its purpose was to ensure that insecticide concentration on or within net fibres remains sufficiently high biological efficacy against susceptible *Anopheles* mosquitoes, after 20 washes. Repeated washing artificially ages nets and is used as a proxy of an ITN used for up to three years under user conditions [19]. Twenty washes indicate chemical durability, simulate real-world handling to improve prediction of field efficacy and product lifespan [8,9]. It can be used to demonstrate polymer–active interactions (diffusion, regeneration, depletion) [4]. Most importantly, artificial aging using 20 washes supports regulatory and procurement decisions [18].

For quality control prior to programmatic distribution, ITNs are evaluated by chemical content. WRI provides a rapid and relatively low-cost measure of batch quality with the aim of predicting wash resistance and overall ITN performance. The CIPAC WRI, defined as the fourth root of the ratio of insecticide content before and after four daily washes, was intended to predict wash resistance (WR) over 20–25 washes. However, our analyses demonstrate that the CIPAC WRI is a poor predictor of WR20, exhibiting a weak and non-proportional relationship, with regression coefficients below 0.5. The principal reason is the disproportionate loss of insecticide during the first wash.

### The First Wash as a Source of Error

Analysis of WHOPES data (2005–2017), supported by experimental results, consistently shows that the first wash removes substantially more insecticide than subsequent washes. Across four polyester nets, the first wash removed 16–27% of insecticide, whereas subsequent washes removed only 3.6–4.1% on average. This early loss reflects storage-and manufacturing-related surface effects rather than intrinsic wash resistance. Including this loss, therefore, compromises prediction of WR20. Excluding the first wash, which captures surface depletion rather than diffusion-limited release, yielded a revised WRI that tracked WR20 more closely.

Because chemical analysis is destructive, the CIPAC WRI methods rely on different samples for pre- and post-wash measurements. This introduces variability due to heterogeneity in insecticide loading, particularly in PET nets [3]. The PermaNet 2.0 WHOPES dataset [20] illustrates this clearly: insecticide content after two washes was lower than after four washes, producing a CIPAC WRI near zero despite New WRI and WR20 values indicating average wash-off of ~3.8%. This aspect can be dealt with by subsampling from a single sample to provide consistent baseline values for comparison.

### Adoption by WHO PQ and Remaining Differences

In 2023, WHO-PQ revised the WRI guidance [18]. In the updated method, the first wash is excluded, but three washes are performed on the first day to establish a standardised starting point analogous to that used in regeneration-time testing, followed by four daily washes. This change in the WHO Guidance is underpinned by a WHOPES appendix that proposed a five-wash procedure [7], although it was not implemented in the 2013 update [6]. The revised WRI is calculated as the ratio of insecticide content after all washes relative to content after the third wash. Although this differs slightly from the approach used here, the underlying principle—removal of first-wash artefacts—is identical. In addition, WHO-PQ guidance mitigates sample heterogeneity by using the same sample for bioassay and chemical analysis and by increasing sample numbers to improve precision. It should be noted that the work presented here is intended as a step towards making WOR predictive. It should be noted that the WHO-PQ revised WRI is used to define a specification that does not claim to be predictive.

### Sampling Variability and Its Consequences

WHO pre-qualification assumes manufacturers can supply representative commercial-scale material. In practice, for donor-funded products, manufacturers seldom produce at scale before recommendation, so small production runs are divided into multiple ‘batches’ for testing. This works reasonably well for polyester (PET) nets, where most variability arises later during coating due to roller performance and fluid pick-up, but it is more problematic for polyethylene (PE) nets. Single-screw extruders used for PE yarns mix poorly, particularly when components melt at different temperatures, so many hours of production are required before the input composition matches the output. Small runs therefore produce heterogeneous yarns. PE nets from pre-qualification batches may therefore show greater variability than PET nets and overcoming this requires evaluation of larger numbers of samples. However, PE nets also release insecticide more slowly, so the ratio of treatment effect to sampling variability is low, further increasing sample size requirements. Once continuous, multi-day production is established, PE nets become substantially more homogeneous. By contrast, polyester (PET) nets are less sensitive in small runs, but their variability may arise later during full-scale coating due to rollers and pick-up.

### From Retention to Loss: WRI versus WOR

Historically, WRI specifications were often manufacturer-defined and occasionally broadened by WHOPES [15], resulting in wide and sometimes uninformative ranges. In some cases, variability even produced WRI values >1, implying an impossible increase in insecticide after washing. Conversely, accepted WRI values as low as 0.8 would imply near-complete insecticide loss (3.5% left) within 15 washes, in practical terms. While the WHO-PQ revision produced narrower and more meaningful specifications, WRI values close to unity (e.g., 0.95–0.99) obscure substantial differences in wash-off. Expressing wash resistance as Wash-Off Rate (WOR) i.e., the per-wash loss, is more intuitive, correlates with surface concentration [4], and aligns more closely with biological endpoints such as mortality. For example, WOR values of 2% and 4% are readily distinguished, whereas WRI values of 98% and 96% may appear deceptively similar.

### Need for a Harmonised Washing Procedure

A single, standardised washing protocol is essential for meaningful comparison. WHO recommended in 2014 that CIPAC soap be used with mechanical shaking [5,6,10] and this remains current best-practice [18]. The washing experiments on PE nets showed no detectable differences in insecticide retention between Marseille and CIPAC soaps. For both deltamethrin and PBO, mean post-wash retention values were similar and no significant detergent effects were observed. This suggests that wash-off in PE nets is governed primarily by polymer chemistry rather than surfactant formulation.

The apparent lack of deltamethrin loss from PE nets washed with Marseille soap was attributable to within-net heterogeneity in insecticide distribution rather than true resistance to wash-off; sampling different sections pre- and post-wash produced post-wash values that were occasionally higher than baseline. To minimise this artefact, multiple subsamples from the same band (measured from the bottom of the net) should be taken, and cutting procedures and sample sizes require careful standardisation to obtain representative estimates of active ingredient loss, particularly when concentrations are low as occurs in incorporated PE nets. Wide net fabrics are more uniform when you look at them in 30 cm strips. The production machines tend to spread the material evenly across these bands due to the intervals of extruders or differences in wear of rollers. In addition, the analytical method must be sufficiently sensitive at low concentrations, as differences in HPLC extraction efficiency or instrumental yield can materially influence the interpretation of wash-off dynamics.

The stability of insecticides on PE is consistent with manufacturing, where actives such as pyrethroids and synergists are incorporated into the polymer prior to extrusion rather than applied as surface coatings, resulting in diffusion-limited release profile that may buffer the insecticide against differential extraction pressures from external detergents. The lack of soap-dependent effects is therefore plausible from a mechanistic standpoint and supports the use of either Marseille or CIPAC soap for wash-testing PE nets in laboratory and semi-field studies. However, a larger investigation is warranted because Marseille soap is still widely used in semi-field trials due to operational challenges and expense of using CIPAC soap in low- and middle-income countries.

### Wash Resistance and Predictive Limits

The Wash Retention Index is intended as a rapid and cost-effective tool used for quality control and product specification, allowing assessment of insecticide retention in ITNs where full wash-resistance testing is impractical. It is used to screen production batches and candidate formulations, support regulatory compliance, and provide an early indication of whether an ITN is likely to retain sufficient insecticide to maintain bio-efficacy prior to deployment.

Although the revised WRI proposed here correlates well with WR20, this does not imply that it predicts insecticide loss under field conditions. Rather, it indicates only that wash-off in well-controlled laboratory wash studies between washes 1–5 is representative of wash-off between 1–20 washes when the wash interval is fixed at one day. Extrapolation from laboratory wash tests to field performance is limited by two issues. First, differences in surface concentration dynamics. Under real-world conditions, long wash intervals permit slow diffusion of insecticide from the polymer interior and recrystallisation (‘blooming’ [21]) leading to the formation of different polymorphs [22] with differing insecticidal activity [23]. This helps explain the wide discrepancies observed between total content and bio-efficacy of ITNs returned from use in the field [24, 25]. Moreover, field decay is only partly attributable to washing; evaporation and abrasion contribute substantially [24,26-29]. Consequently, WR20 tests conducted at 1-day intervals may be insufficient proxies for long-term field durability [19]. Although nets routinely pass laboratory and semi-field wash tests, many fail to kill mosquitoes after less than 2 years of operational use [25,29-31], underscoring the need for durability studies alongside laboratory evaluations.

### Study limitations

The work reported here was unblinded, which could introduce potential biases. The authors have attempted to overcome this by having the chemistry work and the statistical analyses conducted by different individuals. The sample size in the experimental studies is limited, and designed to complement the larger analysis based on literature review. It would be useful to increase the available data on soap washing to improve inter-lab standardisation of chemical methods of ITN surface chemistry, especially because chemical analysis is highly sensitive and ITN chemical specifications are required to be narrow. Evidence for the effect of denier on chemical migration is also limited to coated nets and a larger study including more insecticides as well as PET nets is warranted. In addition, the experimental nets PE with deltamethrin and PBO had the insecticide on separate fibres to allow insecticide release without interaction effects. Fibres were colour-coded for identification. There is a possibility that the dye could affect the migration of insecticide to the surface although, this had been considered in production. The deltamethrin and PBO could be considered as separate products in the statistical model used as it was stratified by insecticide in the effect of soap experiments. There is limited evidence that the addition of dyes in dipped ITNs could affect their efficacy [32]. However, as coloured nets are preferred by many users [33], and are washed less frequently (because they don’t show dirt as soon) the continued use of coloured ITNs should be prioritised, provided there is evidence of their continued efficacy.

## Conclusions

Excluding the first wash from WRI improves prediction of WR20 because the first-wash loss reflects production and storage effects rather than intrinsic wash resistance. The revised WRI can also be calculated using the washing interval derived from chemical analysis of surface concentration, which further improves its correlation with WR20. Although this approach may require slightly more time, it remains substantially faster than a full WR20 study and provides a practical surrogate for estimating insecticidal durability under controlled laboratory conditions. In addition, washing experiments demonstrated no detectable differences in insecticide retention between Marseille and CIPAC soaps for incorporated PE nets, indicating that detergent choice is unlikely to influence wash-off for such products. Wash resistance can be more intuitively expressed as the Wash-Off Rate Index (WORI), representing average wash-off between washes 1-5.
